# Isolation, Characterization, And High Throughput Extracellular Flux Analysis of Mouse Primary Renal Tubular Epithelial Cells

**DOI:** 10.3791/57718

**Published:** 2018-06-20

**Authors:** Wen Ding, Keyvan Yousefi, Lina A. Shehadeh

**Affiliations:** ^1^Department of Molecular and Cellular Pharmacology, University of Miami Leonard M. Miller School of Medicine; ^2^Interdisciplinary Stem Cell Institute, University of Miami Leonard M. Miller School of Medicine; ^3^Department of Medicine, Division of Cardiology, University of Miami Leonard M. Miller School of Medicine; ^4^Vascular Biology Institute, University of Miami Leonard M. Miller School of Medicine; ^5^Peggy and Harold Katz Family Drug Discovery Center, University of Miami Leonard M. Miller School of Medicine

**Keywords:** Biology, Issue 136, Primary cell isolation, primary cell characterization, tubular epithelial cells, kidney, bioenergetics, mitochondria, respiration, high throughput, extracellular flux analysis, fatty acid oxidation, chronic kidney disease

## Abstract

Mitochondrial dysfunction in the renal tubular epithelial cells (TECs) can lead to renal fibrosis, a major cause of chronic kidney disease (CKD). Therefore, assessing mitochondrial function in primary TECs may provide valuable insight into the bioenergetic status of the cells, providing insight into the pathophysiology of CKD. While there are a number of complex protocols available for the isolation and purification of proximal tubules in different species, the field lacks a cost-effective method optimized for tubular cell isolation without the need for purification. Here, we provide an isolation protocol that allows for studies focusing on both primary mouse proximal and distal renal TECs. In addition to cost-effective reagents and minimal animal procedures required in this protocol, the isolated cells maintain high energy levels after isolation and can be sub-cultured up to four passages, allowing for continuous studies. Furthermore, using a high throughput extracellular flux analyzer, we assess the mitochondrial respiration directly in the isolated TECs in a 96-well plate for which we provide recommendations for the optimization of cell density and compound concentration. These observations suggest that this protocol can be used for renal tubular *ex vivo* studies with a consistent, well-standardized production of renal TECs. This protocol may have broader future applications to study mitochondrial dysfunction associated with renal disorders for drug discovery or drug characterization purposes.

**Figure:**
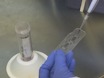


## Introduction

Renal tubular epithelial cell (TEC) function is strongly associated with the overall health condition of the kidney. Pathological signaling in the kidney causes the dedifferentiation of TECs, which plays a major role in kidney fibrosis and chronic kidney disease (CKD)^1^,^2^. As a highly energetic organ, the kidney is second only to the heart in oxygen consumption, primarily through mitochondrial oxidative phosphorylation^3^. Electron microscopy studies have revealed a positive correlation of mitochondrial morphological changes to pathological events in the renal tubules^4^. Mitochondrial dysfunction in TECs causes renal fibrosis through epithelial to mesenchymal transition^5^ and defective fatty acid oxidation^6^. Fibrosis is a progressive renal pathology that results in CKD. Therefore, understanding the energetic status of renal TECs is a necessity to uncover the pathophysiology of CKD.

There are > 20 cell types in the adult kidney^7^. To study the function of TECs, a primary culture of the renal epithelial cells is needed as a platform for molecular biology applications such as chemical treatments and genetic manipulations. Importantly, genetic manipulations can be done *in vivo* in mice via transgenesis or by using AAV gene delivery techniques^8^ so that the isolated primary cells would already be genetically manipulated. The isolation of primary renal tubular cells from mice^9^,^10^, rats^11^,^12^,^13^, canines^14^, rabbits^15^,^16^, and humans^17^,^18^ has been reported with purification steps to yield pure proximal tubular cells. In these previously published protocols that focus on the isolation of proximal tubular cells, gradient centrifugation and sorting experiments were performed for purification purposes^19^. While these protocols are valuable for studying proximal tubules, they are not sufficient when both proximal and distal tubules are needed to be studied. For example, our study on the Alport syndrome has revealed that both proximal and distal renal tubules play important roles in the disease progression^20^, and therefore both kinds of the renal tubules should be investigated in culture. A recent study on renal fluoride toxicity also showed that pathological changes took place in both the proximal and distal tubules^21^. Therefore, this isolation protocol is designed and optimized for both proximal and distal tubular cells from mouse kidneys with a minimal cost of reagents and simple procedures. Alternatively, investigators can still follow the protocol until step 3.1 and add purification steps^9^ from this point forward for the isolation of pure proximal tubular cells.

The isolated cells present high energetic levels and maintain renal epithelial characteristics after the sub-cultures to 4 passages. Using a high throughput extracellular flux analyzer, we assess the mitochondrial respiration directly in the isolated TECs in a 96-well plate, which leads to further insights into cell density optimization. These observations suggest that this protocol can be applied to renal tubular *ex vivo* studies with a consistent, well-standardized production of renal TECs. An added significance of this protocol is its feasible usage as a high throughput tool for the *ex vivo* characterization of mitochondrial bioenergetics in renal proximal and distal tubular cells. Therefore, it can serve as a platform for drug discovery or drug characterization purposes of renal disorders.

## Protocol

All experiments involving animals were approved by the Institutional Animal Care and Use Committee at the University of Miami, conforming to NIH guidelines.

### 1. Plate Coating and Preparation of Reagents

1. Prepare collagen coating:
  1. Add 35 μL of collagen I to 2 mL of a pre-filtered 20 mM acetic acid solution onto a single 60-mm Petri dish. Incubate it at room temperature for 1 h, air-dry it, and expose it to UV.
  2. Wash the coating 3x with PBS to remove any acid residue and save it in a 37 °C CO_2_-free cell culture incubator until the cells are ready for seeding. The final concentration of the collagen coating is 5 μg/cm^2^.
2. Prepare perfusion buffer: add 300 μL of penicillin-streptomycin (P/S) to 30 mL of PBS and warm the mixture up in a 37 °C water bath until the isolation begins.
3. Prepare digestion buffer: dissolve 3.9 mg of collagenase type 2 into 30 mL of PBS, filter the solution through a 0.2-μm bottle-top filter and warm it up in a water bath at 37 °C until the isolation begins.
4. Prepare cell culture media:
  1. Bring the supplements to room temperature. Without filtration, add the supplement (0.05 mL of fetal calf serum, 10 ng/mL of epidermal growth factor, 5 μg/mL of insulin, 0.5 μg/mL of epinephrine, 36 ng/mL of hydrocortisone, 5 μg/mL of transferrin, and 4 pg/mL of triiodo-L-thyronine) to the 500 mL of renal epithelial cell growth basal medium 2.
  2. Warm up the media in a 37 °C water bath until it is ready to use.
5. Prepare compounds: prepare 50 mM FCCP, 10 mM rotenone, 10 mM oligomycin, 10 mM antimycin A, 50 mM L-carnitine, and 50 mM etomoxir stock solutions all in DMSO, aliquot them, and store the compounds at -20 °C.
6. Prepare 2.5 mM sodium palmitate in 220 mL of a 150-mM NaCl solution and warm the solution up in a 75 °C water bath until the palmitate is fully dissolved.
7. Prepare bovine serum albumin (BSA): prepare 0.34 mM fat-free BSA in 250 mL of 150 mM NaCl. The control BSA serves as a negative control for the palm-BSA solution that can be prepared by following step 1.8.
8. Conjugate the palmitate to BSA (Palm-BSA):
  1. Add the palmitate solution gradually into the BSA solution while it is still hot. Then, adjust the pH to 7.4 and mix them at 37 °C for at least 1 h to complete the conjugation.
  2. When the conjugation is completed, add another 150 mL of 150 mM NaCl to the solution, mix it well, and save the aliquots at -20 °C. The final solution contains 1 mM sodium palmitate and 0.17 mM BSA and will be used as a fatty acid substrate for cells in a fatty acid-based extracellular flux assay.
9. Prepare extracellular flux assay basal media:
  1. Add 1 bag of DMEM powder and 20 mL of 200 mM L-glutamine (4 mM final) to 1 L of autoclaved dH_2_O and mix them gently.
  2. On the day of the bioenergetics experiment, add 100 µM sodium pyruvate to the prepared basal media for subsequent preparations of extracellular flux assay media to be used in a glucose- or fatty acid- based respiration assay.
10. Prepare glucose-based media:
  1. To measure cellular respiration capacity through glycolysis, add 17.5 mM glucose powder, 100 µM control BSA (as described in step 1.7), and 20 µM etomoxir (to inhibit the fatty acid oxidation) to the basal media described above in step 1.9.
  2. Warm up the media at 37 °C, adjust the pH to 7.4 and keep it in the 37 °C water bath until it is used in an extracellular flux assay.
11. Prepare fatty acid-based media:
  1. To measure cellular respiration capacity through fatty acid oxidation, add 10 mM 2D-glucose powder (glucose analog to inhibit glycolysis), 100 µM Palm-BSA (as described in step 1.8), and 100 µM L-carnitine to the basal media described above in step 1.9.
  2. Warm up the media at 37 °C, adjust the pH to 7.4, and keep it in the 37 °C water bath until it is used in the extracellular flux assay.

### 2. Perfusion, Digestion and Harvesting Kidneys from Mice

1. Anesthetize the mouse with an isoflurane flow and fix it in a supine position. Make sure the isolation only begins after the animal loses its righting reflexes and the anesthetic depth is monitored by pinch assessments using atraumatic forceps before and during the procedure^22^.
2. Remove fur, using a depilatory cream, from the mouse’s chest to its abdominal area, disinfect it with iodine, and wipe the iodine residue.
3. Make an incision in the chest, cut the skin to open the whole abdominal area, and expose the heart and kidneys.
4. Set up a perfusion pump at 32 mL/min and remove any bubbles in the tubing before starting the perfusion.
5. Insert a 27-G needle into the left ventricle through the heart apex as soon as the buffer fills up the heart, and poke the right atrium to create an exit so the perfusion buffer circulates as the heart pumps and eventually gets removed from the right atrium exit.
6. After the perfusion, switch the pump speed to 30 mL/min for digestion.
7. After 20 mL of digestion buffer is perfused through the apex, remove both kidneys for the tubular cell isolation.

### 3. Tissue Processing and Primary Tubular Cells Isolation

1. Remove the renal capsules and medulla, mince both kidneys into tiny pieces, and incubate them in 10 mL of a digestion buffer in a 37 °C oven with gentle rotation for 5 min.
2. Remove any undigested kidney tissues by passing the buffer through a 70-μm filter. Add 10 mL of culture media to stop the digestion.
3. To collect tubular cells, centrifuge the filtered cell suspension at 50 x g for 5 min to collect the first pellet. Transfer the supernatant to a new tube and add 5 mL of culture media, centrifuge it at 50 x g for 5 min to ensure all tubular cells are collected into the second pellet. The centrifugation is at a lower speed to primarily pellet heavy tubules. Later, after the cells recover from the isolation, the pure tubular culture is centrifuged at a higher speed during the sub-cultures.
4. Resuspend the first pellet in 20 mL of culture media and centrifuge it at 50 x g for 5 min to collect the third pellet.
5. Resuspend the second and third pellets in 1 mL of culture media. Mix 10 µL of the cell suspension with 10 µL of Trypan blue, load the mixture into chamber A of a counting slide, and record the cell viability from the automatic cell counter (see **Table of Materials**).
6. Seed up to 10^7^ cells (a heterogeneous population) onto a single 60-mm dish pre-coated with collagen and let the tubular cells attach overnight.

### 4. Primary Tubular Cells Sub-culture and Characterization

1. On day 1 after the isolation, collect culture media and centrifuge it at 50 x g for 5 min to pellet any floating tubules. Remove the supernatant and resuspend the cell pellet in 4 mL of fresh culture media and plate it back to the same culture dish.
2. On day 4 after the isolation, remove the old culture media and add fresh media.
3. On day 7 after the isolation, detach the cells by incubating them at 37 °C in 2 mL of 0.25% trypsin-EDTA for 5 min. Add 3 mL of culture media to stop the reaction and collect the cells by centrifugation at 250 x g for 5 min.
4. To sub-culture and characterize the cells from P0 to P1, seed 5,000 cells per well onto a 24-well plate coated with collagen I as described above.
5. 24 h after step 4.4, fix the cells at P1 with 4% PFA for 10 min, permeabilize them with 0.2% Triton X-100 for 3 min, and block them with 10% donkey serum (DS) for 1 h at room temperature.
6. Dilute, to 1:100 in 10% DS, each of the following proteins: the proximal tubular markers angiotensinogen (AGT) and aquaporin 1 (AQP1); the distal tubular marker E-cadherin; the mesangial marker CD90/Thy1; and the macrophage markers EGF-like module-containing mucin-like hormone receptor-like 1 (F4/80) and cluster of differentiation 68 (CD68), and incubate them with cells overnight at 4 °C.
7. The next day, detect any staining using 1:200 anti-Rabbit, anti-Mouse, or anti-Rat fluorescent secondary antibodies for 45 min. Take images under confocal microscopy to confirm the expression of the markers, as shown in **Figure 2A**.
8. On day 3 after the sub-culture of P1, detach the cells for a sub-culture and characterization at P2 by a staining of the tubular, mesangial, and macrophage markers described in step 4.5. Image the staining under confocal microscopy to confirm the expression of the markers, as shown in **Figure 2A**.
9. On day 3 after the sub-culture of P2, detach the cells for a sub-culture and characterization at P3 by a staining of the tubular, mesangial, and macrophage markers described in step 4.5. Image the staining under confocal microscopy to confirm the expression of the markers, as shown in **Figure 2A**.
10. Prepare the tissue staining:
  1. Air-dry frozen wild-type and *Col4a3^-/-^* kidney slides for 1 h at room temperature and fix them with 4% PFA for 10 min. Permeabilize them with 0.2% Triton X-100 for 10 min and block them with 10% donkey serum (DS) for 1 h at room temperature. Add antibodies against the marker proteins described in step 4.5 at 1:200 and incubate them at 4 °C overnight.
  2. The next day, detect any staining with 1:200 anti-Rabbit, anti-Mouse, or anti-Rat fluorescent secondary antibodies for 45 min. Take images under a confocal microscope to confirm the expression of the markers, as shown in **Figures 2B **and** 2C**.

### 5. Mitochondria Bioenergetics Assay

1. Seed P1 tubular cells at 20,000, 30,000, or 40,000 cells per well in 100 μL of culture media onto a 96-well microplate pre-coated with 5 μg/cm^2^ collagen I the day before the extracellular flux assays.
2. For the hydration of the sensor cartridge, lift the sensor cartridge and fill each well of the plate with 200 μL of a calibration solution. Carefully load the cartridge back to submerge the sensors in the calibration solution. Place the cartridge in a 37 °C oven without CO_2_ for at least 7 h prior to use. For the best results, overnight cartridge hydration is recommended.
3. Prepare the compounds: prepare 8 µM oligomycin, 9 µM FCCP, and 20 µM rotenone/antimycin A mixture in both glucose (described in step 1.10) and fatty acid (described in step 1.11) extracellular flux assay media.
4. Change the media: aspirate the cell culture media, add 175 µL of glucose or fatty acid assay media (dependent on the compound that is being worked in, see step 5.3), and incubate them for 1 h in a 37 °C CO_2_-free incubator.
5. Load the cartridge ports with 25 µL of the following compounds: 8 μM oligomycin for port A to achieve a final concentration of 1 µM (note: as each well will contain 175 µL of media, the compound will get diluted 8x), 9 μM FCCP in port B to achieve a final concentration of 1 µM (note: as each well will contain 175 µL of media plus 25 µL of the solution injected from port A, the compound will get diluted 9x), and 20 µM rotenone/antimycin A in port C to achieve a final concentration of 2 µM for each compound (note: as each well will contain 175 µL of media plus 50 µL of the solution injected from ports A and B, the compound will get diluted 10x).
6. Add water to all wells in port D and all other ports of the background wells (no cells). Incubate the cartridge in a 37 °C CO_2_-free incubator for 10 min.
7. Turn on the extracellular flux analyzer and the controller.
8. Open the Analyzer Software and input the following protocol:
  1. Choose **Standard Assay**. Press **Assay Wizard**. Using the **Compounds** tab, assign the compound layout and use the **Groups and Labels** tab to label the experimental groups. Remember to assign the empty wells (without cells) as background.
  2. Under the **Protocol** tab, set the following mix and measure cycles using the available commands as indicated in **Table 1**: calibrate, mix for 2 min, wait for 2 min, and measure for 3 min (repeat this cycle 2 - 3x); inject port A, mix for 2 min, wait for 2 min, measure for 3 min (repeat this cycle 2 - 3x); inject port B, mix for 2 min, wait for 2 min, measure for 3 min (repeat this cycle 2 - 3x); inject port C, mix for 2min, wait for 2 min, measure for 3 min (repeat this cycle 2 - 3x). Press **End Wizard**. It is also possible to save the current template for future use.
  3. Press **Start** to begin the calibration. The analyzer then automatically ejects the plate holder and asks for the cartridge plate to be inserted.
9. When the calibration step is done (usually in 20 - 25 min), press the prompt command to change the cartridge plate to the cell plate and continue the run.
10. When the run is completed, transfer the data, and remove the 96-well plate. Add Hoechst (1:1,000) to each of the assay wells and incubate them for 5 min at 37 °C. Normalize the OCR data to cell count by a measurement of the Hoechst fluorescence reading at a 355-nm excitation and a 460-nm emission.

## Representative Results

**Kidney Perfusion and Digestion Yield Highly Viable Tubular Epithelial Cells:** Mouse renal tubular epithelial cells were isolated following the steps outlined in sections 1 - 3 of the protocol described above.

After the digestions, a heterogeneous population of kidney cells, incompletely digested tubules, and other tissue debris that is smaller than 70 µm were plated onto the culture dish on isolation day. Changes from day 0 to day 1 after the isolation are usually expected to be seen only in the cell attachment rather than in the cell growth. Looking through the floating heterogenous population, only a few tubular cells were attached at day 1 (Figure 1A). A re-collection of the cells at day 1, a centrifugation, and a re-plating helped to remove light debris and settle the small tubule pieces for the tubular cell release. From day 1 to day 3, changes were expected not only in a better cell attachment but also in a remarkably improved cell growth rate that was observed with a tripled cell density as compared to day 1 (Figure 1A). In the initial growth phase, the cells formed several colonies and populated around the colonies. From this point forward, the isolated cells were fully recovered and displayed a healthy proliferation. By day 5, the cells were at an 80 - 90% confluency in a 60-mm Petri dish with some spaces between cell to cell and colony to colony (Figure 1A).

**Sub-culture and Characterization of the Isolated Tubular Cells:** The isolated renal tubular epithelial cells were sub-cultured for a characterization following the steps outlined in section 4 of the protocol described above.

From day 5, the cells fully recovered from the isolation and started to proliferate vigorously. One week after the isolation, the cells grew to confluency in a 60-mm Petri dish. After 1 week in a culture at passage 0, the cells were ready to be sub-cultured to passage 1 and, subsequently, for 2 more passages. Similar growth patterns were observed in passage 1 and passage 2. Usually, it takes less than a week for cells at passage 1 and 2 to grow to confluency for further sub-cultures (Figure 1B). The continuous culture of confluent cells from passage 0 to passage 2 showed an extensive dome formation^23^,^24^ (Figure 1B), suggesting that the isolated cells maintained a healthy status where they excreted liquids similar to an *in vivo* status. This caused the monolayer of the cells to lift off the plate but stay connected via tight junctions.

To characterize the cells in the culture, we performed immunofluorescent staining in the cultured cells from passage 1 through passage 4, as well as in a control cell line-human proximal renal epithelial HK-2 cells. Proximal tubular markers, aquaporin 1 (AQP1)^25^ and angiotensinogen (AGT)^9^, the distal tubular marker E-cadherin^25^, the epithelial marker smooth muscle actin (SMA)^26^,^27^,^28^, macrophage markers F4/80^29^ and CD68^30^, and the mesangial marker thymocyte differentiation antigen 1 (Thy1/CD90)^9^ were used for the characterization studies. Both proximal tubular proteins AQP1 and AGT were consistently highly expressed in the isolated tubular cells from passage 1 to passage 4 as well as in the positive control HK-2 proximal epithelial cells (Figure 2A). The distal tubular protein E-cadherin was expressed in the isolated tubular cells through passage 4 and was also observed in the HK-2 cells (Figure 2A). SMA was expressed abundantly in the isolated tubular cells and in the control HK-2 cells, consistent with published reports^26^,^27^. On the other hand, the mesangial protein Thy1 and macrophage protein F4/80 were absent in both the isolated tubular cells and the control HK-2 cells (Figure 2A). CD68 showed a minimal expression in the HK-2 cells and in the isolated tubular cells at passage 1 and passage 2, and then its expression became undetectable from passage 3 through passage 4 (Figure 2A). The results suggest that the cells isolated following this protocol are a mix of proximal and distal tubular cells. To compare the expressions of these marker proteins *in vivo*, we performed staining in frozen kidney tissues. Tubular markers, including AQP1, AGT, and E-cadherin, and mesangial protein Thy1 were found highly expressed in kidneys harvested from a wild-type healthy mouse (Figure 2B). Low expressions of F4/80 and CD68 were observed in wild-type kidneys but extensively expressed in kidneys harvested from a Col4a3^-/-^ mouse that developed renal failure with a macrophage infiltration^20^,^31^ (Figure 2C).

**Mitochondrial Bioenergetics Assay on Isolated Primary Tubular Cells:** The mitochondrial respiration assay steps are outlined in section 5 of the protocol described above.

The mitochondrial respiration of isolated primary tubular cells is measured by an extracellular flux analysis of the oxygen consumption rate (OCR) at different plating densities. To titrate the plating density, 20,000, 30,000, and 40,000 primary TECs per well were seeded onto a 96-well XF96 microplate the day before (approximately 20 h before) the extracellular flux assay (Figure 3A). Following the extracellular flux analysis, the OCR measurements were then normalized to the cell counts by a quantification of Hoechst staining. Plating the TECs at 20,000, 30,000, or 40,000 cells/well resulted in an average basal OCR of 25, 45, or 50 pmol/min, respectively (Figure 3A). Moreover, microscopic images of the plated cells revealed that 40,000 cells/well covered the entire surface of the bottom of the microplate wells better than the other plating densities (Figure 3B). Even though the maximal OCR did not increase using 40,000 cells compared to the density of 30,000 cells, we recommend a cell density of around 40,000 cells/well for optimal interactions between cells and compounds.

Moreover, in our experiments, the optimal concentration of the inhibitory/uncoupler compounds was shown to be 1 µM oligomycin, 1 µM FCCP, and 2 µM rotenone/antimycin-A (the protocols of the extracellular flux assay and the port injections are listed in **Table 1**; the optimization experiments are not shown). However, it is recommended for all users to run a preliminary test with various concentrations of these compounds, ideally lower and higher than the published values, to warrant the best results.

An extracellular flux assay yields a number of important parameters for assessing the bioenergetics of renal TECs. For instance, as fatty acid oxidation is shown to be specifically defective in TECs, the use of media containing fatty acid substrate (palmitate) along with the inhibitor of glycolysis (2DG) can serve as a useful tool to directly evaluate fatty acid oxidation in TECs at passages 1 and 2 (Figure 3C). In the case of renal fibrosis, the overall respiration capacity of the cells is expected to be lower than that of a healthy kidney even though the glucose-based media may not reveal any differences.

Taken together, the extracellular flux assay, especially to assess the fatty acid oxidation capacity, can be utilized as an informative measure to evaluate the energetic status of renal TECs in which pathological changes affecting the bioenergetics profile play a major role in renal fibrosis and the progression to kidney failure.

**Table:** 

Steps	Time (min)
**Calibrate**	
**Equilibrate:**	00:12:00
**Measurement 1**	3 loops
Mix	00:02:00
Wait	00:02:00
Measure	00:03:00
Mix	00:02:00
Wait	00:02:00
Measure	00:03:00
Mix	00:02:00
Wait	00:02:00
Measure	00:03:00
**Inject Port A (1 μM Oligomycin)**	2 loops
Mix	00:02:00
Wait	00:02:00
Measure	00:03:00
Mix	00:02:00
Wait	00:02:00
Measure	00:03:00
**Inject Port B (1 μM FCCP)**	2 loops
Mix	00:02:00
Wait	00:02:00
Measure	00:03:00
Mix	00:02:00
Wait	00:02:00
Measure	00:03:00
**Inject Port C (2 μM Rotenon/Antimycin)**	2 loops
Mix	00:02:00
Wait	00:02:00
Measure	00:03:00
Mix	00:02:00
Wait	00:02:00
Measure	00:03:00

**Table 1. Standardized extracellular flux analysis running protocol for optimal OCR measurements in primary tubular cells.**

**Figure:**
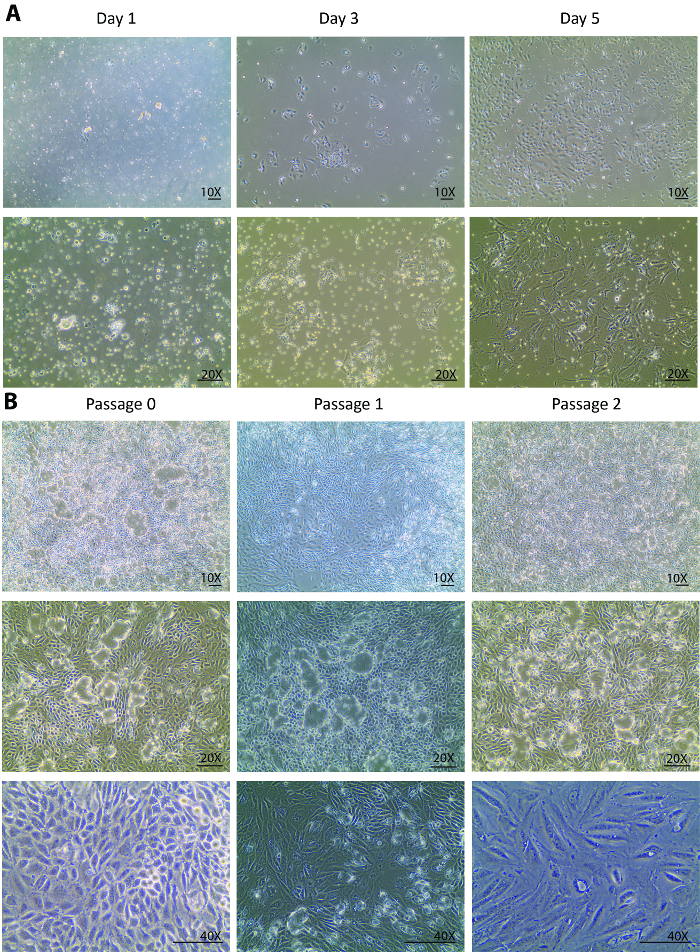

**Figure 1. Cell culture of isolated primary tubular cells.** (**A**) The isolated primary tubular cells attach early at day 1 and grow robustly from day 3 to day 5. The images are taken under 10X and 20X objectives. (**B**) This panel shows a sub-culture of isolated primary tubular cells from passage 0 to passage 3. The images are taken under 10X and 20X objectives for visions of the cell domes, and under a 40X objective for a vision of the morphological changes through the passages. The scalebar = 100 µm. Please click here to view a larger version of this figure.

**Figure:**
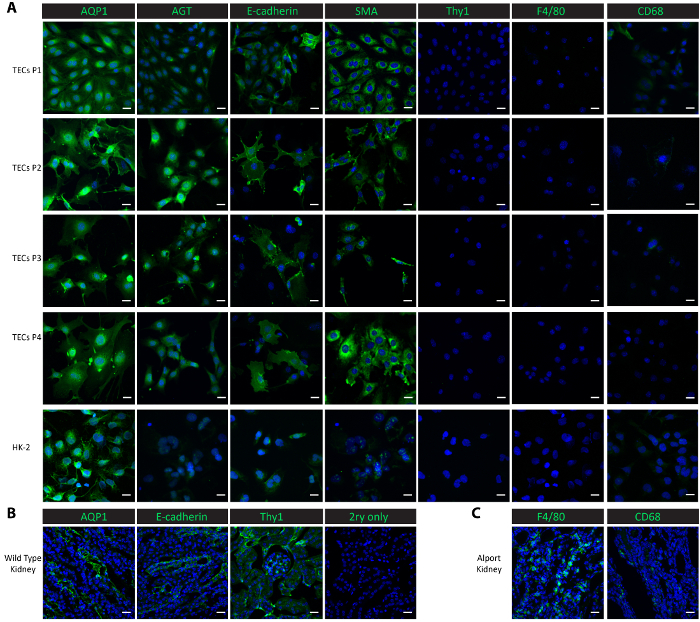

**Figure 2. Characterization of isolated primary tubular cells.** (**A**) Immunostaining with antibodies against proximal tubular proteins (AQP1, AGT), a distal tubular protein (E-cadherin), an epithelial protein (SMA), a mesangial protein (Thy1), and macrophage proteins (F4/80, CD68) show that the isolated primary tubular cells and subsequent sub-cultures are pure proximal and distal tubular cells. (**B**) This panel shows a staining of the proximal tubule, distal tubule, and mesangial proteins in kidney tissues from a healthy wild-type mouse as a positive control and a no-primary negative control. (**C**) This panel shows a staining of the macrophage proteins F4/80 and CD68 in kidney tissue collected from a *Col4a3^-/-^* mouse as a positive control. Scalebar = 20 µm. The DAPI is shown in blue. The marker proteins are shown in green. Please click here to view a larger version of this figure.

**Figure:**
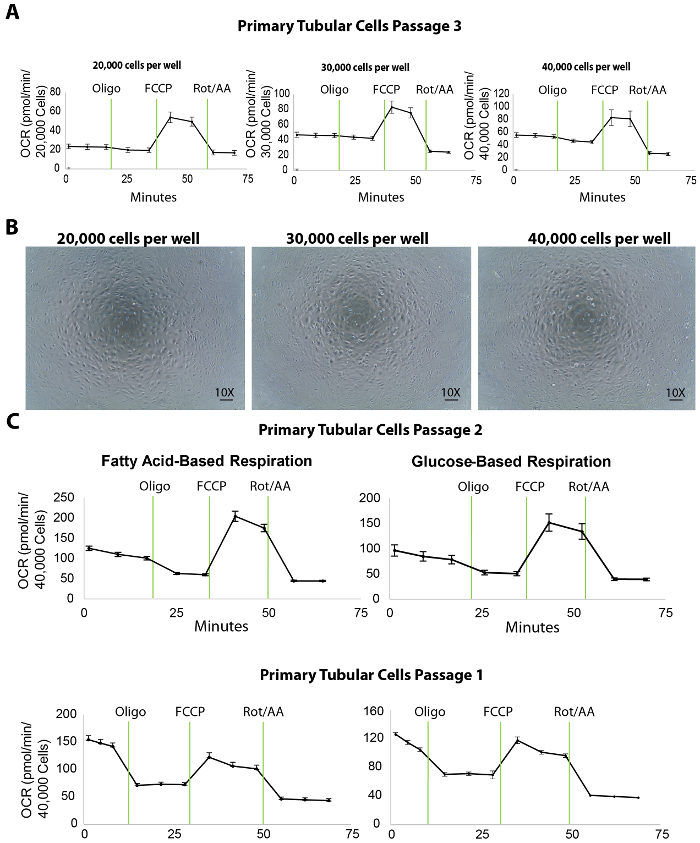

**Figure 3. Extracellular flux assay in isolated primary tubular cells at different plating densities.** (**A**) Increasing cellular plating density enhances the basal respiration levels in primary tubular cells, tested at passage 3. (**B**) This panel shows microscopic images of primary tubular cells cultured on an XF96 microplate seeded at 20,000, 30,000, and 40,000 cells/well densities. Scalebar = 100 µm. (**C**) This panel shows a fatty acid- or glucose-based extracellular flux assay in primary tubular cells at passages 2 and 1. The data is mean ± SEM. Please click here to view a larger version of this figure.

## Discussion

We optimized a protocol that allows for the efficient isolation of mouse renal tubular epithelial cells (TECs) and showed that the cells can be sub-cultured for an extracellular flux analysis to evaluate the mitochondrial respiration in the presence of fatty acid- and/or glucose-based substrates. This protocol is designed for studies focusing on both proximal and distal tubular cells and serves as a framework with which to build more complex experiments for the understanding of TEC pathology-associated kidney diseases. Compared to previously published protocols^9^,^10^,^19^, this method does not require gradient separations with long centrifugation times or an ample antibody usage for sorting and, therefore, offers a more efficient and optimized guide for researchers working in the renal tubular metabolic field. There are several critical steps in this protocol, including the digestion, re-collection, and plating density and compound optimization for the extracellular flux assay.

Choosing the correct type of collagenase and optimal concentration is the key to a successful digestion and dissociation of the tubular cells from renal tissues. Compared to other types of collagenases, type 2 collagenase contains relatively higher levels of protease activity, capable of dissociating compact renal structures. To minimize the chances of contamination as a result of a prolonged perfusion and digestion time, 0.013% type 2 collagenase was perfused at 30 mL/min. The renal capsule was only removed after both kidneys were harvested from the animal and transferred into a sterilized cell culture hood. The kidneys were minced into tiny pieces and continued their incubation with 10 mL of a digestion buffer for another 5 min for a complete digestion and maximal release of the tubular cells.

Although, after the digestion, the tissue suspension is passed through a 70-µm filter to remove very large tissue pieces, there will still be undigested tubules that pass through the filter and stay within the cell suspension and get plated onto the culture dish. It takes a longer time than normal for these tubules to release tubular cells and attach firmly to the culture dish. Therefore, it is rather important to collect the cell suspension and centrifuge it to pellet the unattached tubules and cells the second day after the cell plating. This low-speed centrifugation step further removes other cell types that are lighter than tubular cells and allows for unattached tubules and tubular cells to settle.

The identification of proper cell density is the first and key step for a successful extracellular flux assay. The results showed that 40,000 cells per well on an XF96 microplate is ideal for primary tubular cells in both a fatty acid and a glucose-based respiration assay (Figure 3C). In this protocol, isolated tubular cells were used for the extracellular flux assay at passages 1 and 2. The cells sub-cultured to passage 3, although they maintained an expression of the tubular markers (Figure 2) and a decent performance in the bioenergetics assays (Figure 3A), and showed decreased basal respiration levels compared to passage 2 (shown by comparing OCR in the rightmost panels of Figure 3A to Figure 3C). This decrease may not affect substantially healthy tubular cells (for example, ones isolated from young wild-type mice). However, for studies on cells isolated from CKD mouse models that already have a diminished mitochondrial respiration, higher passages of the cells may cause a further decrease in the basal respiration which would affect the results of the extracellular flux assay. In the studies conducted here, the cells from both passage 1 and passage 2 showed high basal respiration levels. Therefore, following this protocol, we recommend using these two early passages for mitochondrial respiration studies with cells isolated from both healthy and diseased animals. Cells from passage 2 should still be taken into consideration if the passage 1 sub-culture does not yield sufficient cells for the flux assay. In addition to bioenergetics studies, our previous research shows that primary TECs at passage 3 can be extremely useful for treatments with compounds followed by protein and RNA studies (data not shown). That being said, we suggest that investigators using this protocol to isolate tubular cells should carefully choose the optimal passage for different research applications.

The working principle of the extracellular flux analysis is based on the interactions between the injected compounds and the respiration chain complexes and the effect of the uncoupler. Oligomycin is an inhibitor of complex V (ATP synthase) and is used to distinguish ATP-linked oxygen consumption and the oxygen consumption that is required to overcome the regular proton leak across a mitochondrial inner membrane^32^. FCCP uncouples oxygen consumption from the ATP production by disrupting the mitochondrial membrane potential. Thus, it provides a measurement of the maximal respiration capacity as it circumvents the limited capacity of a proton ion efflux by ATP synthase by allowing a proton transport through the membrane. Antimycin-A, a complex III inhibitor, and rotenone, a complex I blocker, are used in combination to shut down the entire mitochondrial respiration allowing a differentiation between the mitochondrial *vs.* the non-mitochondrial oxygen consumption in the cells. These compounds should always be titrated for a specific cell type before the extracellular flux assay to determine the optimal concentrations that yield the optimal OCR curves. Here, we recommend 1 µM of oligomycin, 1 µM of FCCP, and 2 µM of rotenone/antimycin A for the extracellular flux assay on primary TECs.

In conclusion, this protocol provides a simple and cost-effective way to isolate renal primary proximal and distal tubular epithelial cells that can be used for assessing mitochondrial bioenergetics *ex vivo*. While this protocol can be useful in a wide range of molecular biology studies exploring the biological function of renal tubular epithelial cells, we acknowledge its limitations when applying it to studies needing pure proximal or distal tubules. For example, studies on the Lowe Syndrome, a selective proximal tubular dysfunction^33^, or studies on distal renal tubular acidosis, a distal tubular dysfunction^34^, would require a more sophisticated protocol for cell isolation and purification. However, for the majority of the studies that compare tubules *vs.* glomeruli, and for studies to screen potential mitochondrial respiration regulators in tubular cells in general, the protocol provides a feasible high throughput approach. Therefore, this protocol may have broad applications to study mitochondrial dysfunction associated with renal disorders for drug discovery or target validation purposes.

## Disclosures

The authors have nothing to declare.
